# Analysis of Cognitive Skills in History Textbook (Spain-England-Portugal)

**DOI:** 10.3389/fpsyg.2020.521115

**Published:** 2020-11-30

**Authors:** Cosme J. Gómez, Glória Solé, Pedro Miralles, Raquel Sánchez

**Affiliations:** ^1^CEIR Campus Mare Nostrum, University of Murcia, Murcia, Spain; ^2^University of Minho, Braga, Portugal

**Keywords:** history education, textbooks, historical thinking, cognitive skills, epistemology

## Abstract

The main objective of this article is to analyze the cognitive level of the activities in History textbooks in Spain, England, and Portugal in the transition stage from Primary to Secondary Education (11–13 years), according to the country of origin, typology, and the concepts and disciplinary contents included. The design of this research is quantitative, descriptive, and cross-sectional. The non-probabilistic sample consists of 6,561 activities contained in 27 school textbooks from Spain, England, and Portugal. Descriptive and contrast analyses have been carried out using parametric tests. The results indicate that textbooks from Spain and Portugal mainly include activities situated between a basic and intermediate cognitive level while in England, the cognitive level of activities is medium or high. The ANOVA and Tukey *B* tests show significant differences between the cognitive level required in the activities and the typology of exercises, the concepts, and historical contents worked on. The activities with higher cognitive level correspond to those of creation and essays, the exercises that work on empathy and historical relevance, and that contain activities of social and economic history. In contrast, the activities with the lowest cognitive level are short questions and objective tests, those that work on first-order concepts (data and concrete facts), and those on the History of Art. The conclusion is that there is a need for a balanced presence of first-order content and historical thinking skills, the application in the classroom of a more active student-centered methodology, and the teachers’ conception of history teaching that prioritizes historical skills.

## Introduction

The textbook is a cultural, academic, commercial, and ideological product. The purpose of textbooks has always been to bring together the set of fundamental truths that every student must know well and believe; facts, knowledge, and beliefs that are established as closed, immutable, and unquestionable (Viñao, 2003). Carbone (2003) considers that school books are cultural works with their own identity, which must be analyzed from a multidisciplinary point of view. Furthermore, textbooks constitute relevant teaching material that facilitates the learning process and can improve two-way participation between students and teachers. Thus, structured access to this type of attractive material makes lessons more interesting and entertaining (Pavón et al., 2014).

Textbooks for the Spanish curriculum, which is mainly regressive in nature, contain an extension of the curriculum contents so that these reach the classroom with almost no modification-an issue that deserves more attention than it currently receives. In his essay entitled “La historia o la lectura del tiempo,” Chartier (2007) analyzes the debates on History as a narrative story since the 1970s. He highlights works such as that of Certeau (1975), which emphasized the discursive nature of History and how this debate shook the foundations of History as a discipline. The textbook is probably the historical narrative most read by society as a whole, and perhaps, the only history narrative people use throughout their lives, along with other informal means of knowledge (mass media, museums, other centers of historical interest, etc.). This is why it is so important to analyze this story, this narrative, which has created so much controversy through its use (and abuse) in education (Foster and Crawford, 2006).

Despite the continuous legislative changes in the field of education in the last 25 years and the different methodological proposals in the teaching of history, in Spain, the textbook continues to be the main didactic material used by teachers of History (Martínez et al., 2009). Not all the countries around us have the same conception and use of textbooks. While in some countries they are usually used in the classroom to cover a large part of the contents of the subject and to prepare students for the exam, in others, they are just one more resource for both the teaching staff and the student.

The reality is that textbooks fulfill a function of transmitting knowledge and the sense of hegemonic reality on the part of the authorities or of power that is generally not questioned in their pages (Gómez et al., 2014). The textbook, as Foster (2011) indicates, is a powerful cultural artifact containing ideas and values that influential sectors of society expect students to learn and reproduce. In Apple’s words (1993, quoted by Foster, 2011), textbooks are conceived and designed by real people with real interests. Traditionally, the teaching of history has been seen as the means nations use to seek to spread and reinforce the narratives that define the concepts of nation and national identity (Foster, 2011; Miralles et al., 2012). Textbooks contain many of the stories that nations choose to tell about how their institutions, their legitimacy, their relationships with other nations, and the history of their people were built. Therefore, it is the key to analyze the construction of this story along the lines proposed by Chartier (2007) in order to understand the intentionality of the authorities and other agents of influence on the historical narrative that should be present in the classroom.

Concern about the role of the textbook in teaching History and how textbooks are linked to the construction of collective identities has led to a wealth of international literature, such as the special monographic issue of the *International Journal of Historical Learning. Teaching and Research* in 2003, the book compiled by Foster and Crawford (2006), the eBook *O Manual Escolar no Ensino da História: Visões Historiográficas e Didática* coordinated by Solé and Barca (2018), the monographic journal *Educational Inquiry* in 2011 (vol. 2 num. 1), or the monographs in the journals *Ensayos* (2014) and *Historia y Memoria de la Educación* (2017). Articles such as Foster’s (2011) provide an interesting overview of theoretical and empirical approaches to textbook analysis, while Nicholls (2003) or Pingel (2010) present the main methods used in textbook research. However, a key reference is undoubtedly the Georg Eckert Institute for International Textbook Research in Braunschweig (Germany), which is one of the world’s leading textbook research centers with a large number of research projects covering a considerable number of disciplines The Institute conducts multidisciplinary research on textbooks, mainly for the teaching of history and social and cultural studies.

Elmersjö (2014) emphasizes one of the international projects carried out by the Georg Eckert Institute in collaboration with the Swedish universities of Umeå and Karlstad (Gómez et al., 2014). In Europe, therefore, textbook analysis is a fundamental line of research to understand the tensions in the teaching of history, its problems and relations with power, curricula, the construction of identities, and historical, social, and cultural context. Works such as those by Höpken (1996), Cajani (2006), Baquès (2007), Estivalèzes (2011), and Zachos and Michailidou (2014) have gone into these issues in depth, focusing on the construction of the concept of Europe, the presence of the great social and political traumas (First and Second World Wars, fascism, Nazism, etc.), the different ways of approaching the nation, and the changes in the teaching of History visualized in the manuals.

One of the main lines of research in recent decades has centered on the construction of historical thought and what it means to learn skills in the interpretation of the past, beyond conceptual or memorized knowledge (Wineburg, 2001; Van Sledright, 2014). The origins of historical education research in this line are usually set in the 1980s and 1990s (Lee et al., 2004; Lee, 2005). The first works of this project, from the mid-1980s to the mid-1990s, were based on the analysis of students’ historical abilities through the use of historical empathy sources and exercises. More recently, the work of this group has been oriented towards the arguments of students in their explanation of historical processes, combining the management and critique of sources and the different ways of approaching the causality of historical events. In recent years, in the United Kingdom, Chapman (2011) or Cooper (2012, 2013) delve into the arguments of students and their analysis and evaluation of concrete experiences. Elsewhere, Counsell (2011) and Foster (2011) or Harris (2013) focus on the role of historical education in the curriculum, textbooks, and teacher training.

On the other side of the Atlantic, again in the late 1980s and early 1990s, we find the first research works on historical education: Seixas (1993) in Canada or Wineburg (1991) in the USA. The latter used techniques from the field of psychology based on experts and novices (in this case, historians and students) to study what it means to read a historical source and stressed the importance of overcoming presentism. One of the most relevant results of his research is that historical thinking is not a natural ability acquired with psychological development, but requires teaching (Wineburg, 2001).

The research that has influenced the definition of this knowledge of history has multiplied in the 21st century in North America. In the compilation edited by Stearns et al. (2000), with contributions from authors such as Lee, Ashby, Levstik, and Wertsch, there was already a broad reflection on the challenges in the teaching of History at the turn of the century. Barton and Levstik (2004), Levstik and Barton (2008), and Van Sledright (2011, 2014) advanced in the reflection on historical thinking and the development of these second-order concepts. Reisman (2012), Wineburg et al. (2013), and Monte-Sano et al. (2014), among others, examine in depth the skills of the historian and the treatment of sources in the classroom.

Noteworthy in Canada are all the publications by the *Centre for the Study of Historical Consciousness*, directed by Peter Seixas. This center has made a great effort to define historical consciousness and historical thought and to adapt them practically to the reality of the classroom through projects like *Historical Thinking Assessment* (Seixas, 2004, 2017; Lévesque, 2008; Seixas and Morton, 2013; Ercikan and Seixas, 2015). In addition to this center’s efforts, the work on historical education has greatly increased in an attempt to combine the two concepts indicated above: Clark (2011), Létourneau (2014), Zanazanian (2015), or Sandwell and Von Heyking (2014).

In Portugal, Barca (2010), Pinto (2017), Gago (2018), and Solé (2019), and in Spain, López-Facal (2014), Domínguez (2015), Gómez and Miralles (2015, 2016), Sáiz and López-Facal (2015), Gómez and Sáiz (2017), and Carretero (2019) show how proposals from the English-speaking world have been incorporated into research on historical education. In Latin America, historical education research has developed a great deal since the turn of the century and particularly noteworthy is the research being carried out in Mexico and Brazil (Plá, 2005; Schmidt and Fronza, 2018).

## Present Study

In Spain, the analysis of textbooks has been a fruitful line of research over the last 20 years (Valls, 2001, 2007, 2008; Gómez et al., 2014; Ortega et al., 2019). Rafael Valls is the leading Spanish author on this subject and a pioneer in approaching these manuals rigorously and systematically from the didactics of social sciences. From his studies, different themes have been developed in the analysis of textbooks and their relations with the teaching of History in Primary and Secondary Education. The analysis of the contents selected by the manuals is still extremely present. However, this research has been extended to other areas of work such as historical time, the analysis of activities, and their relationship with competences.

Numerous studies have been carried out on textbooks in Portugal in the field of educational history (Matos, 1990; Mendes, 1999) and, more recently, in the field of history education (Magalhães, 2002, 2013; Freitas, 2005; Solé, 2014, 2018; Rodríguez and Solé, 2018; Solé and Barca, 2018). The initial interest was in the analysis of the contents centered on historical themes and on the rigor expressed in them, the analysis of their structure, content, suitability of the methodology, and of the activities and questions posed. Since the 1980s, textbooks have shown greater concern for the nature of history, for making it more objective and scientific, and for a greater rigor in methodological terms (Solé, 2014).

In England, studies on content analysis, often comparative in nature and mainly regarding controversial issues, have been predominant (Grindel, 2012; Gómez and Chapman, 2017).

Due to the paucity of studies on educational competences developed from the activities contained in History textbooks in these countries, this study has focused on the cognitive level of these exercises. The choice of these three countries is due to their different traditions in History education in the case of England which is focused on the development of historical skills, in contrast with Spain’s and Portugal’s emphasis on conceptual contents and transversal competences.

### Research Question, Objectives, and Research Hypothesis

The research question of this paper is: *Are there differences in the cognitive skills demanded in History textbooks in England, Portugal and Spain during the transition from Primary to Secondary education?* The following specific objectives are addressed:

-To analyze the cognitive level of the activities according to the country of origin of the textbooks.

*H*_0:_ There are no statistically significant differences in the cognitive level required by the activities contained in the textbooks of the three countries studied.
*H*_1:_ There are statistically significant differences in the cognitive level required by the activities contained in the textbooks of the three countries studied.

-To analyze the cognitive level of the activities according to the typology of exercises.

*H_0:_* There are no statistically significant differences in the cognitive level of the activities contained in the textbooks according to the typology of the exercises.
*H_1:_* There are statistically significant differences in the cognitive level of the activities contained in the textbooks according to the typology of the exercises.

-To analyze the cognitive level of the activities according to the historical competences they develop.

*H_0:_* There are no statistically significant differences in the cognitive level of the activities contained in the textbooks according to the historical competences developed.
*H_1:_* There are statistically significant differences in the cognitive level of the activities contained in the textbooks according to the historical competences developed.

-To analyze the cognitive level of the activities according to the historical contents that they outline.

*H_0:_* There are no statistically significant differences between the cognitive level required in the textbooks and the historical contents which are presented in these exercises.
*H_1:_* There are statistically significant differences between the cognitive level required in the textbooks and the historical contents which are presented in these exercises.

## Materials and Methods

### Focus of the Research

This research has a descriptive quantitative cross-sectional design, as it seeks to ascertain and compare the characteristics of the activities in the History textbooks of England, Portugal, and Spain. A nominal measurement scale is used to classify the activities according to their typology, competences and historical contents, and an ordinal scale for the cognitive level. The frequency of each of these categories is studied together with the comparison of means between the variables.

### Sample

The sample of this study is made up of the activities included in the Spanish, Portuguese, and English History textbooks for pupils aged between 11 and 13, in the transition period between Primary and Secondary Education. These years correspond in Spain to the 6th year of Primary Education and the first 2 years of Compulsory Secondary Education; in England to the 3 years of Key Stage; and in Portugal to the 6th, 7th, and 8th years. Although the sample is non-probabilistic, nine widely distributed publishers were selected. The publishers are Oxford; Santillana and Vicens Vives in Spain; Heinemann, Hodder Education and Collins in England; and Porto Editora, Asa, and Areal in Portugal. The sample consists of 6,561 activities from 27 school textbooks.^1^

### Data Collection and Analysis

An Excel database was designed to collect the data. The qualitative variables, of nominal type, were coded, assigning them a numerical value to facilitate the subsequent quantitative analysis in SPSS v.22.0. According to the type of activity, the typology of the exercise was defined, employing the classification used by Gómez and Miralles (2015). This categorization is shown in Table 1.

**Table 1:** Type of activities in the textbooks.

Type of activity	Example
Short question	How many dictatorships were there in Spain between 1920 and 1975? What characterizes a dictatorship?
Exercises with figures/images	Working with the image. What differences do you observe between the Christian territories at the beginning of the eleventh century and those in the thirteenth century?
Objective tests	The first Spanish Constitution was passed in: (a) 1808, (b)1812, and (c) 1978 Order chronologically: Caliphate of Cordoba, Taifa kingdoms, Muslim invasion and emirate, Nasrid kingdom of Granada.
Text commentary	Comment on the text. What was the working day of a child in the mines? Could they go to school? What do you think the absence of education meant?
Essay	How did the Jews feel about being expelled? Give reasons for your answer.
Creation	Imagine you are a suffragette at the beginning of the twentieth century. Prepare a poster calling for women’s right to vote. It should include and illustration, a main slogan and three reasons for defending this right.
Information search	In the eleventh century, craftsmen began to form guilds. Search for the main guilds in the Middle Ages and make a list of them.

Source: Own.

We took Sáiz (2013) as our reference in defining the cognitive level required in the activities. Bloom’s taxonomy of learning-objective stages, in one of its most recent versions, adapted by Anderson and Krathwohl (2001), was applied. Other classifications of basic and higher cognitive skills were taken into account (Ramos et al., 2010), as well as the adaptation of exercises on History and Social Sciences to the development of skills (Hernández, 2002; Vidal-Abarca, 2010). This has enabled us to make a hierarchy of cognitive levels of learning resulting from the activities proposed in the textbooks (Table 2). This taxonomy is based on the hierarchy of learning objectives, which include “memorizing” as the initial task, then “understanding”, “applying,” “analyzing,” “evaluating,” and finally, “creating” as the highest level of such objectives. The taxonomy is extremely popular and influential in current educational research and practice (Arievitch, 2020). This review of Bloom’s taxonomy has been used in recent years for the metacognitive analysis of exercises and activities in the field of applied didactics such as the didactics of History, Geography, the experimental sciences and Mathematics (Gómez and Miralles, 2015; Radmehr and Drake, 2018; Virranmäki et al., 2020).

**Table 2:** Meaning and examples of the categorization of the cognitive level demanded of students in the textbook activities.

Cognitive level	Meaning	Example
1	Locating and repeating information in academic texts and primary or secondary written sources. They activate declarative knowledge that is *literal* or *text based*. The only skills required are reading, description, locating, repeating, reproducing, and/or memorizing.	Which foreign countries supported the rebels in the Spanish Civil War?
2	Those that require understanding the information included in the resource (academic text, source, map, chronological axis, image, etc.): summarizing it, paraphrasing it, or schematizing it; locating the main idea of the resource, summarizing the information offered in it and/or schematizing it; defining concepts, relating, establishing similarities, or differences between them; searching for and summarizing new information in other sources; and finally creating simple resources.	How was nineteenth century different from the stratified society?
3	Those that require students to analyze, apply, and evaluate information from different resources or those that involve the creation of new information. They start from the previous level and derive from solving inferential questions and the application of procedural contents as strategies. Exercises of historical empathy, simulations or case studies; the writing of simulated biographies applying the declarative contents learned; and the critical or heuristic evaluation of information provided by the sources.	How do you think your life would be different if there were no democracy in Spain today? Think and explain.

Source: Own, based on the cognitive level categories established by Sáiz (2013).

The study of Seixas and Morton (2013) was taken as the basis for the analysis of the presence of first- and second-order concepts (the latter related to historical thinking skills). The proposal was adapted to the textbook activities, as indicated in Table 3. In addition, two typologies of first-order concepts (chronology; conceptual/factual) were added to the second-order concepts, as has been done in other studies on examinations (Gómez and Miralles, 2015).

**Table 3:** Meaning and example of the categorization of first and second-order concepts in the textbook activities.

Concept	Meaning	Example
Chronology	Knowledge of the dates of historical processes or how to situate them correctly	*Put the following figures in historical order: Charles I, James II, William of Orange, Oliver Cromwell.*
Conceptual/factual	Knowledge of a concept or a specific fact in the past.	*Who were the favorites? Name two of them.*
Historical relevance	Explain the historical relevance of a particular event or figure using appropriate criteria.	*Why was the printing press so important for culture?*
Sources/Historical evidence	Understanding History as an interpretation based on inferences from primary sources.	*Interpretation of texts or sources in the manual that go beyond the reproduction of a sentence.*
Change and continuity	Understanding change in the past as a process, with various rhythms and patterns. Identifying the complex patterns of progress and decadence in different peoples or societies.	*Humanism supposed a change of mentality with respect to the preceding period. Explain its ideas.*
Causes and consequences	Recognizing multiple causes and consequences in the short and long term. Seeing the consequence of a deed or specific person for human activities and current structures and conditions.	*Make a list in your exercise book of the main causes behind the economic crisis of the eighteenth century.*
Historical perspective	Recognizing the differences between current beliefs, values and motivations (world view) and those of earlier peoples and societies. Explaining the perspectives of people in the past within their historical context.	*[regarding an empathy activity with two invented figures from Ancient Egypt] Imagine you are Merit and you wrote a letter to your brother. In your letter explain what the district you live in is like …*
Ethical dimension	Make reasoned ethical judgments about people in the past, taking into account their historical context. Assess the implications for today of the sacrifices and injustices of the past.	*William used terror to try to control the English, but how sensible do you think this was and why?*

Source: Gómez and Miralles, 2016.

The quantitative analysis was performed with SPSS v.22.0. Descriptive statistics were obtained, finding the percentage and absolute frequency of the variables under study. The Kolmogorov-Smirnov test was run to ensure that the sample had normal parameters and that the parametric tests could be performed. Once it was verified that the variables under study complied with these parameters of normality (Sig <0.05), the ANOVA tests between the cognitive level of each one of the exercises with the rest of variables and the Tukey *B* post-hoc test were applied to check the subsets.

## Results

### Cognitive Level by Country of Origin

There is no great difference in the number of activities according to the country of origin (Table 4). In the sample of activities as a whole, the differences between England (*n* = 2,365), Spain (*n* = 2,237), and Portugal (*n* = 1,959) are small. Nevertheless, one important difference is observed at the cognitive level between Spain (1.43) and Portugal (1.44) and that of England (2.44). The Tukey *B* test confirms that the History and Social Sciences textbooks published in Spain and Portugal include activities mostly situated between a basic and intermediate cognitive level, whereas in England, textbooks require students to have an intermediate to high level (Table 4). This difference in means is statistically significant according to the ANOVA test (*p* < 0.05; Table 5).

**Table 4:** Cognitive level by countries and results of Tukey *B* test.

	*N*	Mean	Standard deviation	Tukey *B*^a^^,^^b^ Subset for alpha = 0.05	
				1	2
Spain	2,237	1.43	0.573	1.43	
Portugal	1,959	1.44	0.651	1.44	
England	2,365	2.44	0.587		
Total	6,561	1.80	0.774		2.44

a Using the sample size from the harmonic mean = 2,173.402.

b Group sizes are not equal. Type 1 levels are not guaranteed.

**Table 5:** ANOVA test between cognitive level and country of origin.

	Sum of squares	Gl	Quadratic mean	*F*	Sig.
Between groups	1,546.379	2	773.189	2,130.291	0.000
Within groups	2,380.228	6,558	0.363		
Total	3,926.607	6,560			

### Cognitive Level According to Typology of Question

The difference in the cognitive level of the activities according to the typology of questions is also visible (Table 6). Specifically, the Tukey *B* post-hoc test identifies five homogeneous subsets according to cognitive level and type of activity. The data analyzed show that the activities, which require students to create contents (2.68) or write an essay, require a high cognitive level (2.74). On the other hand, the rest of the activities are associated to a low cognitive level, with the objective tests requiring the lowest cognitive capacity of the students (1.11). It is also evident that the greatest number of activities is associated with types with a low cognitive level, such as short questions (*n* = 2,547) or those that include figures and images (*n* = 1,390), while those linked to a higher cognitive level, such as content creation, are less present in textbooks (*n* = 448; Table 6). The ANOVA test confirms that these differences are statistically significant (*p* < 0.05; Table 7).

**Table 6:** Cognitive level according to typology of activity and results of Tukey *B* test.

	*N*	Mean	Standard deviation	Tukey *B*^a^^,^^b^ Subset for alpha = 0.05				
				1	2	3	4	5
Objective test	434	1.11	0.314	1.11				
Short question	2,547	1.48	0.625		1.48			
Figures/Images	1,390	1.71	0.662			1.71		
Text commentary	711	1.83	0.650				1.83	
Information search	198	1.86	0.761				1.86	
Creation	448	2.68	0.545					2.68
Essay	833	2.74	0.453					2.74
Total	6,561	1.80	0.774					

Table shows the means for the groups in the homogeneous subsets.

a It uses the size of the sample of the harmonic mean = 526.087.

b Group sizes are not equal. The harmonic mean of the group sizes is used.

Type 1 error levels are not guaranteed.

**Table 7:** ANOVA test between cognitive level and type of activity.

	Sum of squares	Gl	Quadratic mean	*F*	Sig.
Between groups	1,562.980	6	260.497	722.320	0.000
Within groups	2,363.626	6,554	0.361		
Total	3,926.607	6,560			

### Cognitive Level According to Historical Thinking Concepts

As far as the cognitive level is concerned, according to the concepts of historical thinking among the eight established categories-two related to first-order and six to second-order concepts-there are notable differences in means (Table 8). The Tukey *B* test establishes five homogeneous subsets that place the categories related to the first-order historical concepts, “Chronology” and “Conceptual/factual” at a low cognitive level (1.28 and 1.32, respectively), while the rest are placed in the intermediate cognitive level, emphasizing the historical “Empathy/Perspective” as the concept that places greatest cognitive level demands on the students, close to level 3 (2.88). In addition, the historical concept that has the most presence in the textbook activities is “Conceptual/factual” (*n* = 2,855) and “Sources/evidence” (*n* = 1,607) while, in contrast, the one that appears least is “Historical Consciousness” (*n* = 143; Table 8). These differences are significant according to the ANOVA test (*p* < 0.05; Table 9).

**Table 8:** Cognitive level according to historical concepts and results of Tukey *B* test.

	*N*	Mean	Standard deviation	Tukey *B*^a^^,^^b^ Subset for alpha = 0.05				
				1	2	3	4	5
Chronology	220	1.28	0.459	1.28				
Conceptual/Factual	2,855	1.32	0.510	1.32				
Sources/Evidence	1,607	2.00	0.716		2.00			
Causes and consequences	498	2.10	0.677		2.10			
Change and continuity	489	2.25	0.687			2.25		
Historical relevance	327	2.44	0.657				2.44	
Historical consciousness	143	2.50	0.592				2.50	
Empathy/Perspective	421	2.88	0.343					
Total	6,560	1.80	0.774					2.88

Table shows the means for the groups in the homogeneous subsets.

a It uses the size of the sample of the harmonic mean = 363.679.

b Group sizes are not equal. The harmonic mean of the group sizes is used.

Type 1 error levels are not guaranteed.

**Table 9:** ANOVA test between cognitive level and historical concepts.

	Sum of squares	Gl	Quadratic mean	*F*	Sig.
Between groups	1,616.146	7	230.878	654.904	0.000
Within groups	2,309.824	6,552	0.353		
Total	3,925.970	6,559			

### Cognitive Level According to the Typology of the History Content

Finally, in the relationship between cognitive levels and the typology of History contents, there is also a difference in means, albeit not as high as in the other categories (Table 10). The results indicate that political and institutional History is the most worked on in the activities (*n* = 2,986) followed by social and economic History (*n* = 2,290). On the other hand are the contents related to History of Art and Culture (*n* = 1,022) and General History (*n* = 262). Social and economic history activities are quite close to an intermediate cognitive level (1.96), while activities related to contents on the history of art and culture have the lowest cognitive level (1.54) according to the Tukey *B post-hoc* test (Table 10). The ANOVA test confirms that these differences are statistically significant (*p* < 0.05; Table 11).

**Table 10:** Cognitive level cognitive according to historical contents and results of Tukey *B* test.

	*N*	Mean	Standard deviation	Tukey *B*^a^^,^^b^ Subset for alpha = 0.05		
				1	2	3
History of art and cultural	1,022	1.54	0.644	1.54		
General history	262	1.73	0.771		1.73	
Political and institutional history	2,986	1.77	0.782		1.77	
Social and economic History	2,290	1.96	0.779			1.96
Total	6,560	1.80	0.774			

Table shows the means for the groups in the homogeneous subsets.

a It uses the size of the sample of the harmonic mean = 718,540.

b Group sizes are not equal. The harmonic mean of the group sizes is used.

Type 1 error levels are not guaranteed.

**Table 11:** ANOVA test between cognitive level and historical contents.

	Sum of squares	Gl	Quadratic mean	*F*	Sig.
Between groups	133.303	3	44.434	76.809	0.000
Within groups	3,792.667	6,556	0.579		
Total	3,925.970	6,559			

## Discussion and Conclusion

After analyzing more than 6,500 textbook activities from three different countries, we cannot reject the alternative hypotheses: there are statistically significant differences between the cognitive level of the activities and the countries of origin, the type of exercises, the historical competences that they develop, and the contents proposed. With this analysis, it is possible to deduce which exercises require a greater or lesser cognitive effort on the part of the pupils. Essay-writing and creative activities which work on empathy, perspective and historical consciousness, and which develop social and economic history contents, are those which present a greater cognitive level (moderate to high). On the other hand, short questions and objective tests focusing on chronology and first-order contents (facts and concepts), and which develop history of art contents, are those which present a lower cognitive level. While textbooks from England employ the former typology of activities, Spanish and Portuguese manuals contain a higher percentage of activities with a low cognitive level.

Among the main causes for this difference between the three countries, the different curricular approaches and epistemological conceptions of the teaching of history stand out. In England, the historical competences (empathy, historical argumentation, causes and consequences, etc.) are prescriptive elements of the curriculum. There is a recommended list of first-order contents (facts, reigns, concepts, etc.), although they are not obligatory. These first-order contents are the elements which make it possible to develop the previously mentioned competences. On the other hand, in Portugal and Spain, there is a list of first-order contents which are for prescriptive learning, the inheritance of a model of General History. This approach to the teaching of History was defined in the 19th century and aimed to teach all historical events chronologically, be they of a nation/State or the whole of humanity (Valls and Colomer, 2018). This model is primarily based on the national narrative which is accepted and promoted by the institutions of power and which normally eclipses all other alternative narratives (López-Facal, 2014; Ender, 2019). The greater freedom of the English curriculum allows textbooks to contain a greater number of essay-writing and creative activities regarding contents of social and economic History, whereas, although these types of activities exist in Portuguese and Spanish textbooks, these focus more on the knowledge (mainly based on memorization) of characters, events, and reigns related with the institutions of power and with a national and European narrative (Carretero and Van Alphen, 2014; Gómez and Chapman, 2017).

The different approach of the English curriculum is the consequence of the remarkable impact of projects such as *Concepts History and Teaching Approaches* (CHATA) and the changes introduced by the *National Curriculum* of 1991 (Cooper and Chapman, 2009; Byrom, 2013). The CHATA project, which continued into the early years of the 21st century, was based on the acquisition of second-order concepts by pupils and on the establishment of levels of progression of this knowledge in ages ranging from 7 to 14 (Domínguez, 2015). In this regard, the contributions made since the 1980s by a large number of authors (Lee et al., 2004; Lee, 2005) are of great interest as they advocated a form of history teaching which included, in a balanced way, both conceptual and procedural contents, which they began to call second-order concepts (Martínez-Hita and Gómez, 2018).

In Spain, Ley Orgánica (8/2013), for the improvement of educational quality provides the framework for Real Decreto (126/2014), 28th of February, which establishes the basic curriculum of Primary Education (pupils aged 6–11), and Real Decreto (1115/2014), 26th of December, which establishes the basic curriculum of Compulsory Secondary Education and Baccalaureate (pupils aged 12–17). In both educational stages, the programming of objectives, contents and evaluation criteria, and evaluable learning standards are centered on the transmission of disciplinary theoretical knowledge, with there being very little of significance on the teaching of skills that require active learning methods and the continuous evaluation of the learner. Indeed, the main criticisms are the large volume of theoretical content and the excessive number of evaluable learning standards, which condition a teaching model centered on the teacher and characterized by the predominance of the master class and the final assessment of the students (López-Facal, 2014).

In Portugal, the *Currículo Nacional do Ensino Básico* (6–15 years) and *Ensino Secundario* (16 to 18 years) Currículo Nacional - DL (139/2012) regulated the education system until its reform in 2018. Criticism of the Portuguese curriculum has been quite similar to that in Spain both in terms of the extension of content programs and the traditional teaching approach. Indeed, the Curricular Goals introduced in education legislation in 2011/12 (Ministério da Educação e Ciência (MEC-DGS), 2013) are similar to the evaluable learning standards of the Spanish curriculum. In both cases, the student learning process is centered on the reproduction of contents acquired fundamentally through memorization, and consequently, the teaching-learning process revolves around a low cognitive level. The latest legal review in Portugal incorporates the document Aprendizagens Essencias, Despacho no. 6944-A (2018) dated 19 July 2018, which goes beyond disciplinary contents, that is, significant, indispensable, and relevant knowledge, and seeks to value the abilities and attitudes that students must develop. In other words, it again values teaching in which the competences are recognized as indispensable. It deals with what students must know, the cognitive processes they must activate in order to acquire this knowledge, and also the know-how, or the transformation, of this knowledge into new learning. It is oriented towards the *Perfil dos Alunos à Saída da Escolaridade Obrigatória*, Despacho no. 6478 (2017), dated 26 July 2017. The document incorporates contributions on teaching and learning from international reference bodies (the European Union, the Organization for Economic Cooperation and Development and the United Nations Educational, Scientific and Cultural Organization), as well as recent research in education and curricular documents from other countries.

The high presence of objective tests and short questions in Spain and Portugal do not require comprehension tasks on the part of the pupils nor the application of new knowledge; consequently, they are at a low cognitive level. This type of activity is also frequently used in student assessment instruments (Trepat, 2011; Gómez and Miralles, 2015; Merchán, 2015), which corroborates the fact that in countries like Spain and Portugal (despite the changes made in the latter since 2017) priority is given to the reproduction of memorized content, with no process of critical reflection or significant learning.

It is true that the analysis of primary and secondary sources is present in all the curricula of the countries analyzed, as is reflected in the high number of activities related to the commentary of texts or the inclusion of figures and images. However, this widespread presence in the activities of History and Social Sciences books does not imply a high cognitive level, as in most cases, these sources have an illustrative function and are not used to encourage students to ask questions about them, or to compare information and draw conclusions that confirm or modify the initial hypotheses regarding a historical fact. In short, the activities that could put pupils in contact with the scientific method of History as a discipline, with the job of the historian, are limited to the demand for a descriptive analysis of the same, which is of little use in learning historical skills (Sáiz, 2014). This has been found in other European countries such as France (Van Nieuwenhuyse, 2016) and Holland (Kleppe, 2010). For all these reasons, the activities in History and Social Sciences textbooks that include the presence of texts, figures, or images are at a low cognitive level.

This way of conceiving History gives greater importance to political events, giving the discipline a marked positivist bias that we can still find today in Spanish and Portuguese textbooks of History and Social Sciences, and which is demonstrated by the high number of activities focusing on political and institutional content. The contents of Social and Economic History, with fewer contents and activities, allow publishers to deploy a rich repertoire of images, figures, and graphic designs that make the textbooks visually appealing. Art and culture appear have a low presence in all the books analyzed, mainly as support material, as they do in the state curricula, and therefore, their function in the activities is limited to descriptive tasks of the proposed sources.

In contrast, the search for information, the creation of contents, and the writing of essays are at an intermediate to high cognitive level. This type of activity has been found, above all, in History textbooks from England, where, from the early stages of education, pupils are encouraged to reflect critically on historical phenomena and facts from sources, using strategies of inquiry (project work, problem-based learning, etc.) and simulation (role playing, gamification, etc.).

The cognitive level demanded by the activities analyzed in Spanish and Portuguese textbooks is low. This is not only the case when comparing them with English textbooks. There are other studies on the cognitive level of textbooks and examinations using this revised version of Bloom’s taxonomy which show significant differences with the results found. One example are the intermediate accounting textbooks, which only contain 10% of activities of cognitive level 1 (memorization and reproduction of contents), compared with 62% of application and analysis exercises (Davidson and Baldwin, 2005). There are also differences with the Finnish Geography examinations analyzed, in which 28% of the exercises was of level 1, compared to 72% of comprehension, application, and creation activities (Virranmäki et al., 2020). Furthermore, there are differences with the analysis of digital textbooks carried out by Hung Lau et al. (2018). The approach of the curriculum and the hegemony of a model of History education based on the national narrative are an obstacle to the cognitive demands of the activities of History textbooks.

The curriculum is therefore fundamental when it comes to improving the teaching of history. The insertion of second-order concepts or historical competences, together with the traditional first-order concepts, is fundamental if students are to learn to think historically and be citizens who are critical of the world around them. The last reform carried out in Portugal with the incorporation into the curriculum of the Aprendizagens Essenciais, Despacho no. 6944-A (2018) of 19 July 2018, is situated in this line. It constitutes a shift towards a more Anglo-Saxon approach to teaching, centered on teaching second-order historical concepts related to historical thinking. In addition, greater flexibility in content programming and greater investment in schools have led Portugal to significantly improve its PISA test score in recent years. This is not the case of Spain, whose most recent educational reform continues to be anchored in a traditional teaching approach, with an excessive content curriculum, a history teacher training that retains strong deficiencies and a public school network in need of material and human resources. Perhaps the greatest changes in the medium term can be seen in the area of initial teacher training, due to the improvements made to the curricula in Spanish universities to include subjects related to the teaching of the Social Sciences. In addition, there is the challenge of connecting the scientific environment of universities, where pioneering research on innovative methods and strategies for learning about history is carried out, with educational centers in order to achieve a transfer of knowledge that represents a significant improvement in History teaching.

## Limitations and Future Research

Among the limitations of this paper are that we can point out the unidimensionality of the source studied and the need to combine quantitative and qualitative methods. Furthermore, this paper does not delve into the use that the teacher makes of the textbook. Not all teachers use the textbook in the same way. There is a margin of professional autonomy when deciding how to use these materials. Therefore, this study analyzes the suitability of textbooks according to the countries of origin and the type of exercises they contain. But it does not delve into the use that teachers make of these textbooks and the possible differences according to the countries or the level of training of the teachers.

New lines of research must go this way. The new concept of a textbook should be studied, as it becomes a bank of audiovisual and multimedia resources. In this paper, we have analyzed the student textbook. But publishers for a few years have been providing teachers with other online resources. A new research challenge is to compare these online resources for teachers. It would also be necessary to analyze the use of the textbook by students and teachers: good practices and the effectiveness of its use in the conceptual change in students and their preconceptions and ideas. Finally, the degree of acceptance of the manuals among the teachers according to their previous training and their methodological and epistemological conceptions of history should also be analyzed.

To advance in these lines of research, a combination of sources and quantitative and qualitative approaches would be necessary. In addition to the analysis of the textbook exercises, interviews and discussion groups with teachers and students should be carried out. In this combination of sources, the use and preference of textbook exercises by teachers and students should be investigated on the basis of the sociodemographic and predictive variables indicated above.

## Data Availability Statement

The datasets generated for this study are available on request to the corresponding author.

## Author Contributions

CG and PM: conceptualization, methodology, validation, funding acquisition, and resources. CG and GS: software, investigation, and data curation. CG, GS, PM, and RS: formal analysis, writing-review, and editing. CG: writing-original draft preparation. CG, GS, and RS: visualization. PM: supervision and project administration. All authors contributed to the article and approved the submitted version.

### Conflict of Interest

The authors declare that the research was conducted in the absence of any commercial or financial relationships that could be construed as a potential conflict of interest.

## Footnotes

^1^We thank María Martínez Hita because she entered half of the activities of textbooks of England and Spain in the database on the framework of it Final Master’s Thesis. Also we thank Arthur Chapman for pointing out the most suitable English textbooks for analysis.
